# Depression and Associated Factors among Community-Dwelling Thai Older Adults in Northern Thailand: The Relationship between History of Fall and Geriatric Depression

**DOI:** 10.3390/ijerph191710574

**Published:** 2022-08-25

**Authors:** Thin Nyein Nyein Aung, Saiyud Moolphate, Yuka Koyanagi, Chaisiri Angkurawaranon, Siripen Supakankunti, Motoyuki Yuasa, Myo Nyein Aung

**Affiliations:** 1 Department of Family Medicine, Faculty of Medicine, Chiang Mai University, Chiang Mai 50200, Thailand; 2 Global Health and Chronic Conditions Research Group, Chiang Mai University, Chiang Mai 50200, Thailand; 3 Department of Public Health, Faculty of Science and Technology, Chiang Mai Rajabhat University, Chiang Mai 50300, Thailand; 4 Department of Medical and Health Science, Tokyo Ariake University, Tokyo 135-0063, Japan; 5 Centre of Excellence for Health Economics, Faculty of Economics, Chulalongkorn University, Bangkok 10330, Thailand; 6 Department of Global Health Research, Graduate School of Medicine, Juntendo University, Tokyo 113-8421, Japan; 7 Faculty of International Liberal Arts, Juntendo University, Tokyo 113-8421, Japan; 8 Advanced Research Institute for Health Sciences, Juntendo University, Tokyo 113-8421, Japan

**Keywords:** aging, community-integrated intermediary care (CIIC), geriatric depression, fall, older adult, Thailand

## Abstract

Background: Globally, population aging is happening more quickly than in the past, and Thailand ranks the world’s number three among the rapidly aging countries. Age-related decline in physical and mental health would impact depression among older adults. We aimed to determine the depression among the community-dwelling Thai older adults in Chiang Mai, Thailand. Methods: The baseline data, collected by door-to-door household visits of an intervention arm from a cluster randomized controlled trial (Community-Integrated Intermediary Care (CIIC): TCTR20190412004), were included in this cross-sectional study. Descriptive analysis and binary logistic regression were applied. Results: The mean age was 69.31 ± 7.10 years and 23.8% of study participants were older than 75 years. The Thai geriatric depression scale showed 6.5% had depression. Adjusted risk factors for depression were older age, being single, drinking alcohol daily, having diabetes, having experience of a fall last year, self-rated health as neutral, poor/very poor, and moderate/severe dependency by ADL scoring. Conclusion: Our findings highlighted the potentially modifiable risk factors in addition to the common predictors affecting depression among community-dwelling older adults. Fall prevention programs and public health interventions to prevent diabetes are recommended. Furthermore, self-rated health and Barthel’s ADL scoring would be simple tools to predict risk factors for geriatric depression.

## 1. Introduction

Globally, population aging is happening more quickly than in the past, with 22% of the total global population estimated to be aged 60 years and above by 2050 [1,2]. According to the World Health Organization, 80% of older people will be living in low- and middle-income countries in 2050, and such developing nations with limited resources to prepare for long-term care may experience the severe impact of population aging. Although Thailand has been upgraded to the upper middle-income country, it ranks the world’s number three in the speed of population aging. It is also the fastest population aging country in Southeast Asia, experiencing rapid aging with 20% of its population aged sixty and over (about 8.5 million people aged 60 years and above). This demographic change may challenge the government’s fiscal burden, especially the rising welfare budget, health care needs, and long-term care costs for the aging population [3,4].

Age-related physical changes and social changes would impact the mental health of older adults. Approximately 15% of older adults suffer from mental disorders and, amongst them, depression is the most common mental disorder, affecting 7% of the world’s elder population [5]. Depression is the leading cause of disability worldwide which is related to the considerable morbidity and premature mortality, and also increases the perception of poor health, utilization of health care services, and costs [6]. It is estimated that 1.5 million Thai people suffer from depression which is the number one cause of years of life loss due to disability among females in 2013 [7]. According to the Thailand national health survey 2010, depression was prevalent among 9.3% of the population aged 60 years and above. Depression results from a complex interaction of social, psychological, and biological factors and the effect of depression can be long lasting or recurrent. The seventh elderly population survey in Thailand showed that the northern region of Thailand has the highest percentage of senior citizens (above 25.2%) and Chiang Mai is one of the provinces listed in the top 5 for the largest number of aged people [3]. The northern region ranked second to the central and eastern region for the high risk of suicide among depressed Thai older adults, 1.58% and 2.40%, respectively, in 2017 [8,9].

Like other Asian settings, intergenerational coresidence and the concept of filial reciprocity remains an integral part of informal caregiving for older adults in the Thai context [10,11]. Material support, social support, personal assistance, and care provided by adult children prevail in taking care of elderly Thai people and family-based long-term care is currently the prevalent living arrangement of older adults [12]. However, due to the changing demographic context, socioeconomic environment, family structure, and migration of adult children for job opportunities, the traditional care of elderly people at home in their community is being challenged. Social ties are important for the moral support of older adults and social network diversity is negatively associated with geriatric depression [13,14]. The current prevalence and factors associated with depression among community-dwelling older adults may be different from those found in earlier research [15,16,17,18] due to the changes in social livelihoods and a higher proportion of dependency than in the past. Therefore, we aimed to determine the prevalence and associated factors of depression among Thai community-dwelling older adults using the geriatric depression scale (GDS).

## 2. Materials and Methods

### 2.1. Data Collection and Participants

This study was conducted in accordance with the Declaration of Helsinki. Ethical approval was given by the World Health Organization Ethical Review Committee: WHO/ERC ID; ERC.0003064 and Ethical Review Committee for Research in Human Subjects: Boromarajonani College of Nursing Nakhon Lampang: Praboromarajchnok, Institute for Health Workforce Development, Ministry of Public Health, Thailand; E 2562/005. It was also registered at Thailand Clinical Trial Registry (TCTR20190412004).

This study was a subgroup analysis of baseline characteristics of the participants from an intervention arm of a cluster randomized controlled trial, Maehia subdistrict, Mueang Chiang Mai, Thailand. The sample size and power estimation were carried out by using STATA version 11SE (Stata Corporation, College Station, TX, USA) and it was aimed to recruit 2000 participants in each arm. Persons aged 60 years and above, either male or female and residents in the study site, were recruited with their written informed consent. Unconsenting people and persons with cognitive impairment were excluded. According to the CIIC study protocol, the data were collected via door-to-door visits for six months, using interviewer-administered survey questionnaires by well-trained research assistants in 2019 [19].

### 2.2. Measures

The structured questionnaires included sociodemographic characteristics, health behaviors, underlying diseases, history of fall last year, dependency, and depression assessments. The sociodemographic factors included age (completed years), gender, marital status (single, married, or divorced/separated/widow), education (completed primary school or completed secondary school and above), residential type (residence of the original village or housing estate), staying alone or with family, and type of caregivers (children/grandchildren, spouse, siblings, or others). Health behaviors included smoking habits (current smokers or not), exercise status (exercise or not), and alcohol drinking habits (current daily drinkers, never drank, already quit, or occasional drinkers). Underlying diseases such as hypertension, diabetes mellitus (type 2), and dyslipidemia, and having history of fall in the last year were explored.

Dependency was assessed by the Barthel index of activities of daily living (ADL) which is widely used for assessing the current level of fundamental daily life activities of older adults such as feeding, grooming, bathing, dressing, bowel and bladder care, toilet use, ambulation, transfer, and stair climbing. It is commonly used by Thai researchers and validated in the Thai setting [20]. ADL total score ranged from 0 to 20, and it was categorized into two groups: 0–11 moderate to severely dependent and ≥12 mildly dependent to independent elderly participants.

Depression was measured by the Thai 15-item geriatric depression scale (GDS). The 15-item GDS is a standardized tool which is commonly used to assess the level of depression in Thailand [21]. It consists of 15 items with “yes” or “no” responses. Each response is scored either as 0 points or 1 point, and total points are summed up to obtain the GDS total score, ranging from 0 to 15. A dichotomous scale of having depression (total GDS score 6–15) and no depression (total GDS score 0–5) was made to determine the depression among study participants [18,22,23].

The GDS and ADL scales are well validated and widely used in Thailand. Following the WHO process of research instrument translation [24], we also checked the overall reliability of the research instruments in our study setting and found them to be reliable with Cronbach’s alpha of GDS and ADL of 0.80, 0.90, respectively.

### 2.3. Data Analysis

SPSS version 22 (IBM Corporation, Armonk, NY, USA) was used for data analysis. Sociodemographic characteristics were analyzed by descriptive analysis. The dependent variable in this analysis was having depression (total GDS score 6–15). The factors affecting depression were determined by binary logistic regression with factors with a *p* value of ≤0.05 with a 95% confidence interval (CI) regarded as significant factors.

## 3. Results

### 3.1. Sample Characteristics

In total, 1509 participants were included in the final analysis. The mean age was 69.31 ± 7.10 years (60–99 years) and nearly a quarter of them were old-old (23.4%). The study population comprised women (61.8%), married people (59.8%), people educated at least at the primary level (61.6%), and original village community residents (80.8%); 6.1% were staying alone and 93.9% with family, with children/grandchildren (46.1%) being the most common coresidents for elder persons. Ninety-seven percent of participants were nonsmokers, 67.1% never drank, and 77.8% exercised regularly. The most frequent comorbidity was hypertension (46.8%), followed by dyslipidemia (21.5%), and diabetes (17.6%). Of participants, 14.3% had experience of a fall in the last year, 2.3% of participants were moderately to severely dependent in terms of Barthel’s ADL scores, and 48.2% rated their health as good/very good. The baseline characteristics of the study participants are described in Table 1.

### 3.2. Prevalence and Factors Associated with Geriatric Depression

The geriatric depression scale showed 6.5% of older adults had depression. Sociodemographic factors significantly affecting depression were old-old (adjusted odds ratio (adjOR) 2.03, 95% confidence interval (95% CI) 1.23–3.35) and being single (adjOR 2.97, 95% CI 1.41–6.28) when compared to young-old and married counterparts, respectively. We did not find any significant associations between gender, education, residential type, or staying alone and depression.

Regarding health behaviors, daily alcohol drinkers had depression 3.19 times more often than other participants who never drank alcohol (adjOR 3.19, 95% CI 1.44–7.07). Smoking status and exercise habits did not have any significant association with depression in this study.

Having diabetes was found to be a comorbid condition which significantly affected depression, as diabetic participants had depression 2.22 times more often than nondiabetic counterparts (adjOR 2.22, 95% CI 1.28–3.84), whereas other underlying diseases such as hypertension and dyslipidemia were not associated with depression. Seniors with history of a fall in the last year were 2.34 times more likely to have depression than those who had no experience of a fall (adjOR 2.34, 95% CI 1.41–3.88). Self-perceived health status reflected the depression prevalence and those who rated their health as neutral (2.26 times) (adjOR 2.26, 95% CI 1.27–4.00) and as poor/very poor (7.87 times) (adjOR 7.87, 95% CI 3.64–16.98) were more likely to have depression than participants who rated their health status as good/very good. Moderately to severely dependent study participants (ADL less than 12) were 5.31 times more likely to have depression than independent to mildly dependent participants (adjOR 5.31, 95% CI 2.16–13.08). The results are summarized in Table 2.

## 4. Discussion

This study reported the depression among community-dwelling older adults and its predictors. Previous studies in Thailand reported that the prevalence of depression among community-dwelling older adults varied from 10.3% to 43.0% [13,25,26,27]. Of our study participants, 6.5% had depression which was lower than in the preexisting studies. The difference in prevalence of depression could be caused by the different regions of study site and the different measurement tools. Our study participants being mainly composed of the residents of an original village community with a stronger social network could be an explanation for this lower prevalence of depression.

Advanced age has been hypothesized to be a risk factor for depression among older adults. However, depression is not a normal part of aging, and is a treatable medical condition. Many factors associated with the prevalence of mental disorders such as painful life events, comorbidities, and past depression may worsen when they become old [28,29,30]. Similar to another study of community-dwelling elderly people in Japan, depression increased two times amongst the old-old participants compared to young-old participants [31]. Contrary to prior studies, the present study did not show that female gender, smoking habit, and exercise were related with depression [27,32,33].

The study participants who drink alcohol daily were significantly associated with depression, more than three times, when compared to nondrinkers. The combination of depression and problems related to alcohol consumption in older adults increases the potential for poor mental and physical health outcomes [33]. Depression is an expected reaction to the loss of loved ones and marital status is one of the significant factors affecting depression. However, we noted that single participants had a prevalence of depression three times higher than married participants, similar to another Thai study [34]. Being single as a risk factor for depression in older adults could explain this finding [35].

Consistent with other studies, the study participants who had a history of a fall in the last year were more prone to have depression, 2.35 times more, than their counterparts. Depression is considered as one of the most common risk factors for falls and subjects who fall are more prone to anxiety and depression. Depression, fear of falling, and experience of a fall are considerably interconnected and have a significant bilateral relationship [36,37,38]. Depression was significantly associated with physical disability (ADL) in later life. The study participants who were functionally dependent in terms of Barthel’s activities of daily living scoring had depression 5.3 times more often than functionally independent individuals. This finding was consistent with other studies where depression in old age was found to be an independent risk factor for disability; similarly, disability is a risk factor for depression [39,40].

Our study participants self-rated their health as poor/very poor (6.0%) and as neutral (45.8%) which is comparable with another study of elderly Thai people where 56% of the participants rated their health as either fair or bad/very bad. Psychosocial symptoms have significant effects on older persons’ perception concerning their own health and our finding of self-rated health and its association with depression supported this study [41]. Inevitable age-related physical and psychosocial changes can have effects on both diabetes and depression among older adults. Depression may affect and be affected by diabetes. Depression directly impairs the ability to produce or use insulin through hormonal, neuronal, and immune system changes. Moreover, depression indirectly negatively affects self-care behaviors such as overeating, drinking alcohol, or nonadherence to diabetes medications and follow-up appointments. Prolonged exposure to psychosocial stressors of having a chronic medical condition, such as diabetes and its related complications, makes diabetic patients prone to have depression [42,43]. The finding of a significant association of diabetes with depression in our study supported other studies where diabetes was associated with prevalent and incident depression [42,43,44]. Coexistence of diabetes and depression is associated with increased morbidity and mortality and a subsequent increase in health care costs. Efforts to identify and treat depression among diabetic older adults should be encouraged. Notably, falls and diabetes are potentially modifiable risk factors. The older adult population could be screened to identify these risk factors and these individuals should be targeted for intervention to abate such modifiable risk factors and cause a subsequent reduction in risk of depression.

## 5. Limitations and Strengths of the Study

Most study participants were residents of an original village community where the rich and poor households are in close contact with strong social ties. This could have impacted on the prevalence and predictors of depression when compared to the residents of private housing estates in gated communities. Moreover, due to the cross-sectional nature of the study, causality cannot be determined and the findings from older adults of the northern region may not be representative of other regions of Thailand. Familial factors which may have important impacts on geriatric depression were not included in this study. However, reaching the 1509 community-dwelling Thai older adult participants by door-to-door visits for about 6 months before the COVID-19 pandemic was a great opportunity to determine their preexisting geriatric depression and it strengthened the findings of our study.

## 6. Conclusions

Our findings highlighted the potentially modifiable risk factors in addition to the common predictors affecting depression of community-dwelling older adults which can be applicable for the potential interventions of a topic often overlooked as a simple part of aging. Fall prevention programs are recommended to reduce the dependency and associated depression. Moreover, the association of having diabetes with depression highlighted the need for public health intervention not only to prevent the increasing burden of noncommunicable diseases but also to improve the mental health of older adults. The assessment of self-rated health and screening of dependency using Barthel’s ADL scoring are simple useful tools for the early detection of such a common geriatric disease which could be of importance in preparing for aging in places with active and healthy aging communities.

## Figures and Tables

**Table 1:** Sociodemographic characteristics of study participants, Maehia, Chiang Mai, Thailand (*n* = 1509).

Elderly Participants	Depression		
	Yes	No	Total
	*n* (%) *	*n* (%)	*n* (%)
Total population	98 (6.5)	1411 (93.5)	1509 (100%)
Age			
<75 years	56 (57.1)	1100 (78.0)	1156 (76.6)
≥75 years	42 (42.9)	311 (22.0)	353 (23.4)
Gender			
Male	34 (34.7)	543 (38.5)	577 (38.2)
Female	64 (65.3)	868 (61.5)	932 (61.8)
Residential type			
Housing estate	14 (14.3)	275 (19.5)	289 (19.2)
Original community	84 (85.7)	1136 (80.5)	1220 (80.8)
Marital status			
Married	47 (48.0)	856 (60.7)	903 (59.8)
Single	13 (13.3)	103 (7.3)	116 (7.7)
Separated, divorced, widowed	38 (38.8)	452 (32.0)	490 (32.5)
Education			
Secondary school and above	23 (23.5)	556 (39.4)	579 (38.4)
Primary school completed	75 (76.5)	855 (60.6)	930 (61.6)
Staying alone			
No	94 (95.9)	1323 (93.8)	1417 (93.9)
Yes	4 (4.1)	88 (6.2)	92 (6.1)
Types of caregivers			
Son, daughter, grandchildren	52 (53.1)	644 (45.6)	696 (46.1)
Spouse	31 (31.6)	553 (39.2)	584 (38.7)
Siblings	7 (7.1)	61 (4.3)	68 (4.5)
Others (relatives, maid, friends)	8 (8.2)	153 (10.8)	161 (10.7)
Current smoking			
No	96 (98.0)	1360 (96.4)	1456 (96.5)
Yes	2 (2.0)	51 (3.6)	53 (3.5)
Drinking			
Never	68 (69.4)	944 (66.9)	1012 (67.1)
Quit	14 (14.3)	189 (13.4)	203 (13.5)
Occasional	4 (4.1)	176 (12.5)	80 (11.9)
Daily	12 (12.2)	102 (7.2)	114 (7.6)
Exercise habit			
Exercise	61 (62.2)	1113 (78.9)	1174 (77.8)
No exercise	37 (37.8)	298 (21.1)	335 (22.2)
Underlying diseases			
Hypertension			
No	49 (50.0)	754 (53.4)	803 (53.2)
Yes	49 (50.0)	657 (46.6)	706 (46.8)
Diabetes			
No	67 (68.4)	1176 (83.3)	1243 (82.4)
Yes	31 (31.6)	235 (16.7)	266 (17.6)
Hyperlipidemia			
No	69 (70.4)	1116 (79.1)	1185 (78.5)
Yes	29 (29.6)	295 (20.9)	324 (21.5)
History of fall last year			
No	65 (66.3)	1228 (87.0)	1293 (85.7)
Yes	33 (33.7)	183 (13.0)	216 (14.3)
Self-perceived health			
Good/very good	19 (19.4)	708 (50.2)	727 (48.2)
Neutral	52 (53.1)	639 (45.3)	691 (45.8)
Poor/very poor	27 (27.6)	64 (4.5)	91 (6.0)
Barthel’s activities of daily living			
≥12	82 (83.7)	1393 (98.7)	1475 (97.7)
<12	16 (16.3)	18 (1.3)	34 (2.3)

* Column percentage ‡.

**Table 2:** Factors associated with depression among study participants, Maehia, Chiang Mai, Thailand (*n* = 1509).

Demography	Depression			
	Frequency (%) ^‡^	Adjusted OR	95% Confidence Interval	
			Lower	Upper
Age				
<75 years	56 (4.8)	Referent		
≥75 years	42 (11.9)	2.03 *	1.23	3.35
Gender				
Male	34 (5.9)	Referent		
Female	64 (6.9)	1.04	0.59	1.84
Residential type				
Original community	84 (6.9)	Referent		
Housing estate	14 (4.8)	0.97	0.49	1.92
Marital status				
Married	47 (5.2)	Referent		
Single	13 (11.2)	2.97 *	1.41	6.28
Separated, divorced, widowed	38 (7.8)	0.96	0.56	1.65
Education				
Secondary school and above	23 (4.0)	Referent		
Primary school completed	75 (8.1)	1.48	0.85	2.55
Staying alone				
No	94 (6.6)	Referent		
Yes	4 (4.3)	0.65	0.21	1.98
Current smoking				
No	96 (6.6)	Referent		
Yes	2 (3.8)	0.47	0.10	2.21
Drinking				
Never	68 (6.7)	Referent		
Quit	14 (6.9)	0.83	0.40	1.71
Occasional	4 (2.2)	0.59	0.19	1.81
Daily	12 (10.5)	3.19 *	1.44	7.07
Exercise habit				
Exercise	61 (5.2)	Referent		
No exercise	37 (11.0)	1.07	0.63	1.81
Hypertension				
No	49 (6.1)	Referent		
Yes	49 (6.9)	0.64	0.38	1.07
Diabetes				
No	67 (5.4)	Referent		
Yes	31 (11.7)	2.22 *	1.28	3.84
Hyperlipidemia				
No	69 (5.8)	Referent		
Yes	29 (9.0)	1.32	0.76	2.27
History of fall last year				
No	65 (5.0)	Referent		
Yes	33 (15.3)	2.34 *	1.41	3.88
Self-perceived health				
Good/very good	19 (2.6)	Referent		
Neutral	52 (7.5)	2.26 *	1.27	4.00
Poor/very poor	27 (29.7)	7.87 **	3.64	16.98
Barthel’s activities of daily living				
≥12	82 (5.6)	Referent		
<12	16 (47.1)	5.31 **	2.16	13.08

* *p* value < 0.05, ** *p* value < 0.01, ^‡^ row percentage.

## Data Availability

The data presented in this study are available on request from the corresponding author. The data are not publicly available because this study was a sub-group analysis of baseline data of the intervention clusters from a cluster randomized trial and the final analysis is ongoing and publications of the whole cluster randomized trial are not yet finished.
